# Antioxidant airway responses following experimental exposure to wood smoke in man

**DOI:** 10.1186/1743-8977-7-21

**Published:** 2010-08-20

**Authors:** Maria Sehlstedt, Rosamund Dove, Christoffer Boman, Joakim Pagels, Erik Swietlicki, Jakob Löndahl, Roger Westerholm, Jenny Bosson, Stefan Barath, Annelie F Behndig, Jamshid Pourazar, Thomas Sandström, Ian S Mudway, Anders Blomberg

**Affiliations:** 1 Department of Public Health and Clinical Medicine, Division of Medicine, Umeå University, Umeå, Sweden; 2 Division of Respiratory Medicine and Allergy, Department of Medicine, University Hospital, Umeå, Sweden; 3 Kings College London, MRC-HPA Centre for Environment and Health, School of Biomedical and Healthy Studies, King's College London, London, UK; 4 Energy Technology and Thermal Process Chemistry, Umeå University, Umeå, Sweden; 5 Division of Aerosol Technology, Lund University, Lund, Sweden; 6 Department of Physics, Lund University, Lund, Sweden; 7 Department of Analytical Chemistry, Arrhenius Laboratory, Stockholm University, Stockholm, Sweden

## Abstract

**Background:**

Biomass combustion contributes to the production of ambient particulate matter (PM) in rural environments as well as urban settings, but relatively little is known about the health effects of these emissions. The aim of this study was therefore to characterize airway responses in humans exposed to wood smoke PM under controlled conditions. Nineteen healthy volunteers were exposed to both wood smoke, at a particulate matter (PM_2.5_) concentration of 224 ± 22 μg/m^3^, and filtered air for three hours with intermittent exercise. The wood smoke was generated employing an experimental set-up with an adjustable wood pellet boiler system under incomplete combustion. Symptoms, lung function, and exhaled NO were measured over exposures, with bronchoscopy performed 24 h post-exposure for characterisation of airway inflammatory and antioxidant responses in airway lavages.

**Results:**

Glutathione (GSH) concentrations were enhanced in bronchoalveolar lavage (BAL) after wood smoke exposure vs. air (p = 0.025), together with an increase in upper airway symptoms. Neither lung function, exhaled NO nor systemic nor airway inflammatory parameters in BAL and bronchial mucosal biopsies were significantly affected.

**Conclusions:**

Exposure of healthy subjects to wood smoke, derived from an experimental wood pellet boiler operating under incomplete combustion conditions with PM emissions dominated by organic matter, caused an increase in mucosal symptoms and GSH in the alveolar respiratory tract lining fluids but no acute airway inflammatory responses. We contend that this response reflects a mobilisation of GSH to the air-lung interface, consistent with a protective adaptation to the investigated wood smoke exposure.

## Background

Within the European Community there is an increasing commitment for replacing the present dependence on fossil fuel energy sources with renewable CO_2_-neutral alternatives. By 2020, the member states of the European Union are committed to a target of 20% of their energy requirements from renewable sources, including biomass. Although the scheme supports many types of renewable heat, special concern has been expressed to the urban air pollution health impact presently arising from traditional wood log installations, and the potential increase in new small and medium scale biomass heating systems [1-4]. Combustion-derived particulate matter (PM) air pollution has been widely considered to be associated with adverse respiratory and cardiovascular health effects [3,4]. In many European countries, residential biomass combustion contributes substantially to the fine particulate emissions (PM_2.5_), which has also been confirmed in other parts of the world including the US where a range of 8-85% has been given [1,5-8]. The emissions are mainly due to the use of present old and non-optimised, small biomass combustion systems.

The negative impact on health of biomass combustion has been demonstrated by the higher occurrence of respiratory symptoms and respiratory-related hospital admissions, among populations living in areas with a high wood smoke-derived contribution to ambient PM [3,9-14]. Asthmatic subjects have been shown to be particularly sensitive to wood smoke [10,12,15].

To date, only a few experimental studies have investigated the toxicity of wood smoke derived PM to compliment the epidemiological observations. Cell culture studies have shown that exposure to wood smoke particles results in the release of pro-inflammatory cytokines, affects cell viability and induces oxidative stress and DNA damage [3,16,17]. These findings are to some extent similar to those reported after in vitro challenges of airway cells to diesel exhaust-derived and other ambient air pollution particles [18-21]. Further, the first human in vivo study examining the effects of wood smoke has recently been reported [22-24]. The investigators employed batch wise firing of log wood in a cast iron stove with healthy subjects exposed to diluted wood smoke at PM_2.5 _concentration of about 250 μg/m^3 ^for four hours. This was associated with minor increases in amyloid A protein and altered coagulation factor ratios in blood, increased exhaled nitric oxide and oxidative stress reflected by increased malondialdehyde in exhaled breath condensate [22-24].

Whilst these papers have relied on indirect measures of airway inflammation and oxidative stress, the present study was performed employing bronchoscopy based lavage which may give a clearer picture of the responses occurring at the air-lung interface following wood smoke inhalation. The primary hypothesis was that exposure to wood smoke would cause an inflammatory response in human airways, triggered through the imposition of oxidative stress, as has been demonstrated in humans following diesel exhaust challenges [25-29].

## Methods

### Subjects

Nineteen healthy, non-smoking non-allergic volunteers (10 males/9 females) with a mean age of 24 years (range 21-31 years) were recruited. All had negative skin prick tests against a standard panel of aero-allergens and normal lung function. All subjects refrained from medication, including vitamin or antioxidant supplementation for the duration of the study and were free from symptoms of airway infection for a six week period prior the wood smoke and air exposures. The local ethics committee (Umeå University, Umeå, Sweden) approved the study and all participants gave their informed written consent. The study was conducted according to the declaration of Helsinki.

### Exposure

Subjects were exposed on two different occasions in a randomised, double blinded fashion; once to filtered air and once to diluted wood smoke-derived emissions in a purpose-built exposure chamber (18 m^2^, suitable for 1-2 persons) at Umeå University, with an interval of at least three weeks. Each exposure lasted for three hours, during which the subjects alternated between 15-minute intervals of exercise on a bicycle ergometer (minute ventilation 20 L min^-1 ^m^-2 ^body surface) and rest. The filtered air, with or without diluted biomass combustion emissions was introduced through the ceiling by air distributors and were driven by a slight overpressure through openings at floor level to an antechamber. The residence time of the filtered air/wood smoke in the exposure chamber was approximately 6 minutes.

### Combustion system and exposure conditions

The biomass combustion emissions were generated using a typical residential wood pellet burner (nominal effect 15 kW) installed in a reference boiler. A moist softwood pellet/sawdust fuel mixture from pine and spruce (18% moisture) was burnt under adjusted combustion conditions, i.e. low temperature (700-800°C) and reduced air/fuel mixing, to simulate wood smoke emissions from incomplete combustion. The combustion conditions were monitored by measuring O_2 _and CO concentrations in the flue gases, as well as the temperature in the combustion zone. The incomplete combustion conditions resulted in flue gas (i.e. undiluted emissions) with CO concentrations of 1000-5000 ppm and O_2 _levels of 6-11%. A fraction of the emissions was subsequently cooled and diluted using a three steps dilution system to the desired PM concentration. Measurements of gases and particles in the chamber were performed in order to monitor and control the exposure conditions. The flue gases were diluted 200-300 times with an average air-exchanged rate of 10/h during the exposures. The temperature in the chamber was maintained between 21-24°C with a relative humidity 20-50%.

The concentrations of CO and NO_x _(dominated by NO) in the chamber were monitored continuously and varied in the range of 5-15 ppm and 0.2-0.4 ppm, respectively. The concentration of two volatile organic compounds, i.e.1.3-butadiene and benzene, were determined during five exposures by SKC Ultra passive (diffusive) samplers and gas chromatography analysis. The concentrations of 1.3-butadiene and benzene were 11.0 ± 2.8 μg/m^3 ^and 81.8 ± 14.3 μg/m^3^, respectively. The fine particle mass concentration in the chamber was monitored by a Tapered Element Oscillating Microbalance (TEOM 1400) equipped with a PM_2.5 _pre-cyclone. The operation temperature of the TEOM was set to 30°C, instead of the standard of 50°C, which aimed at reducing potential evaporation of particulate organic material from the filter. To avoid water condensation at this lower temperature, a Nafion dryer (Perma Pure LLC) was used after the cyclone but before the TEOM. The fine PM in the chamber varied in the range of 180-300 μg/m^3 ^during each exposure with an average of the mean concentration from all exposures of 224 ± 22 μg/m^3^.

The particle mass size distribution in the chamber was measured with a 13 stage low-pressure cascade impactor in the range of 0.03-10 μm (aerodynamic diameter) during two exposure occasions. The mass median diameter (MMD_a_) of the fine PM was 0.218 μm and 0.228 μm during these two occasions. The fine particle number size distribution and concentration in the chamber was measured during five exposure occasions by a scanning mobility particle sizer (SMPS) system, in the range of 0.016 to 1.00 μm (equivalent mobility diameter). The particle number concentration varied in the range of 5-10 ×10^4 ^cm^-3 ^with a mean concentration of 6.7 ± 0.9 × 10^4 ^cm^-3^. The geometric mean diameter (GMD_m_) was 0.120 ± 0.018 μm (mean ± SD), with a geometric standard deviation (σ_g_) of the size distribution of 1.81 ± 0.06. However, a log-normal mode fitting calculation showed that the number size distribution comprised two distinct modes (mode peak diameter at 0.100 and 0.190 μm, respectively) which show that the wood smoke aerosol was an external mixture of two different particle types. Corresponding measurements in the filtered air used for the "clean air" exposures showed a particle number concentration of 1-2 cm^-3^.

The PM was characterized chemically regarding carbon fractionation in organic and elemental carbon (OC/EC), major inorganic ions, trace elements and polycyclic aromatic hydrocarbons (PAH).

The fraction of OC and EC of the PM in the chamber was determined by thermal-optical carbon analysis (Method NIOSH 5040) using quartz and teflon+quartz filters in parallel according to standard procedures [30]. A relatively high OC/EC ratio was determined for the present wood smoke PM. Major inorganic ions were also analyzed by ion chromatography (IC) after ultrasonification of the Teflon filter samples collected by the OC/EC measurements. The dominating ions were K^+^, SO_4_^2-^, CO_3_^2- ^and Cl^-^, i.e. alkali salts like K_2_SO_4_, K_2_CO_3 _and KCl. The PM in the chamber was also sampled on specific polycarbonate filters for analysis of trace elements (Z > 12) by Particle-Induced X-ray Emission (PIXE) technique. Zn was the dominating trace element followed by Mo, Rb and Pb. Thus, an overall estimated chemical fractionation of the used diluted wood smoke revealed that the PM consisted of 60% OC, 25% EC, 13% alkali salts and 2% trace elements.

Further, measurements of PAH in the chamber was performed during three exposure occasions by sampling with glass fibre filters for the particulate fraction followed by a polyurethane foam (PUF) plug for the semi-volatile fraction. 45 specific PAH compounds were analyzed by gas chromatography-mass spectrometry (GC-MS), according to procedures described previously [31]. The total PAH (particulate and semi-volatile) concentration was 757 ± 167 ng/m^3 ^where 74-88% was semi-volatile. Dominating PAH's in the semi-volatile fractions were Phenanthrene and Fluorene followed by Anthracene, Fluoranthene, Pyrene and Methylphenanthrenes. In the particulate fraction Benzo(b)fluoranthene totally dominated, followed by e.g. Fluoranthene, Benzo(e)pyrene, Pyrene, Cyclopenta(cd)pyrene, Benzo(c)phenanthrene, Phenanthrene and Benzo(a)pyrene.

### Assessment of lung function and symptom perception

Lung function tests including vital capacity (VC), forced vital capacity (FVC) and forced expiratory capacity in one second (FEV_1_), were performed pre-exposure as well as immediately after, 4 and 24 hours post-exposure using a Vitalograph^® ^spirometer (Buckingham, UK). The perceived intensity of a range of symptoms was recorded by the subjects at seven different time points; before, every 30 minutes during the exposure and immediately after each exposure. The symptoms were scored with a ranking from 0 (no symptoms) to a maximum of 11, according to a modified Borg scale [32], as previously reported [33]. The protocol included questions of unpleasant smell or taste, eye, nasal or throat irritation, cough, nausea, headache, difficulty in breathing, tiredness and chest tightness.

### The fraction of exhaled nitric oxide

FE_NO _concentrations were evaluated pre exposure, immediately after, 4 and 24 hours post-exposure, using a nitric oxide analyzer (NIOX^®^, Aerocrine AB, Stockholm, Sweden). Four different exhalation flow rates; 10, 50, 100 and 270 mL×s^-1 ^(± 10%), during a slow exhalation against an oral pressure of 2 cm H_2_O for 25, 10, 8 and 6 seconds respectively were examined. The measurements were conducted in triplicate at each flow rate and the mean concentration of exhaled NO (ppb) was registered according to ERS/ATS guidelines [34].

### Airway lavage based analyses

Bronchoscopy was performed 24 hours post-exposure using a flexible video bronchoscope (Olympus BF IT160; Olympus, Tokyo, Japan), as previously described [26,29]. Bronchial wash (BW) with 2 × 20 ml and bronchoalveolar lavage (BAL) with 3 × 60 ml sterile phosphate-buffered saline (PBS) (pH 7.3, 37°C) was performed. The BW and BAL samples were collected into separate siliconised containers and immediately placed on ice. They were then filtered through a nylon filter (pore diameter 100 μm; Syntab AB, Malmö, Sweden) and centrifuged at 400 g for 15 minutes. Cell pellets derived from the first bronchial (BWI) and the pooled BAL aspirates were resuspended in PBS at a cell concentration of 10^6 ^cells/ml and differential cell counts were performed on cytocentrifuge slides stained with May-Grünwald Giemsa, counting 400 cells per slide [26,29]. The phagoctyic activation/degranulation markers myeloperoxidase (MPO) (BioCheck, Inc., CA, USA) and matrixmetalloproteinase 9 (MMP-9) (R&D Systems, Inc., MN, USA) were analysed in the cell free lavage supernatants derived from the first BW fraction and BAL, using commercially available enzyme linked immunosorbent assay kits.

### Antioxidant analysis

Cell-free BW (second fraction) and BAL supernatants were analysed for total protein, glutathione (GSH and glutathione disulphide (GSSG)), vitamin C (ascorbate and dehydroascorbate), and urate concentration. Total protein was measured by reaction with bicinchoninic acid and 4% copper (II) sulphate, following the method of Smith et al. [35]. Total glutathione concentrations were measured using the GSSG-reductase-DTNB recycling method [36,37]. Ascorbate and urate were measured simultaneously by reverse phase HPLC with electrochemical detection, as previously described [38]. Total vitamin C (dehydroascorbate + ascorbate) was measured by pre-treating samples with 50 mM Tris(2-carboxylethyl) phosphine for 15 minutes to reduce oxidised ascorbate and then performing the lipid extraction and HPLC analysis as described above. The dehydroascorbate concentration was then calculated by subtracting the ascorbate concentration from the total vitamin C concentration.

Endobronchial biopsies were also taken during bronchoscopy and snap-frozen before storage at -80°C. Biopsies were then homogenised and analysed for their total protein, glutathione, ascorbate and urate concentrations, as described previously, as well as their heme oxygenase 1 (HO-1) protein concentration and glutathione S-transferase (GST) activity. Biopsies were thawed on ice prior to homogenisation and placed in 100 mM sodium phosphate extraction buffer (pH 7.5). Zirconium beads were added and vortexed for 5 minutes to disrupt the cells and the resulting homogenate used for analysis. HO-1 protein concentration was measured by an ELISA from Stressgen Reagents (Ann Arbor, MI, USA), and the glutathione S-transferase activity measured using an assay kit from Sigma Aldrich (Poole, UK, catalogue number CS0410) and employed 1-Chloro-2,4-dinitrobenzene (CDNB) as a substrate as it is suitable for the broadest range of GST isozymes.

### Statistical analysis

All biomedical analyses were carried out in pairs with the subject acting as his/her own control; comparing air exposure data to the corresponding time-point data collected following wood smoke exposure. The symptom data were analysed using the non-parametric Kruskal-Wallis test. Lung function, FE_NO _and peripheral blood cell counts were analysed using a repeated measures ANOVA, with data presented as mean +/- standard error of mean (SEM). The comparison of airway inflammatory and antioxidant markers between the two exposures were calculated using the non-parametric Wilcoxon's Signed Rank test, with data presented as median and IQR (inter quartile range). The correlation analyses were carried out using the Spearman-Rank test. In all cases, a significant difference was assumed at the 5% level. All statistical analyses were performed using SPSS^® ^version 17.0 (SPSS inc., Chicago, IL, USA).

## Results

All subjects tolerated the exposures and bronchoscopy procedures well, with equivalent bronchial and bronchoalveolar lavage recoveries from all 19 subjects post air and wood smoke exposure.

**Figure 1:**
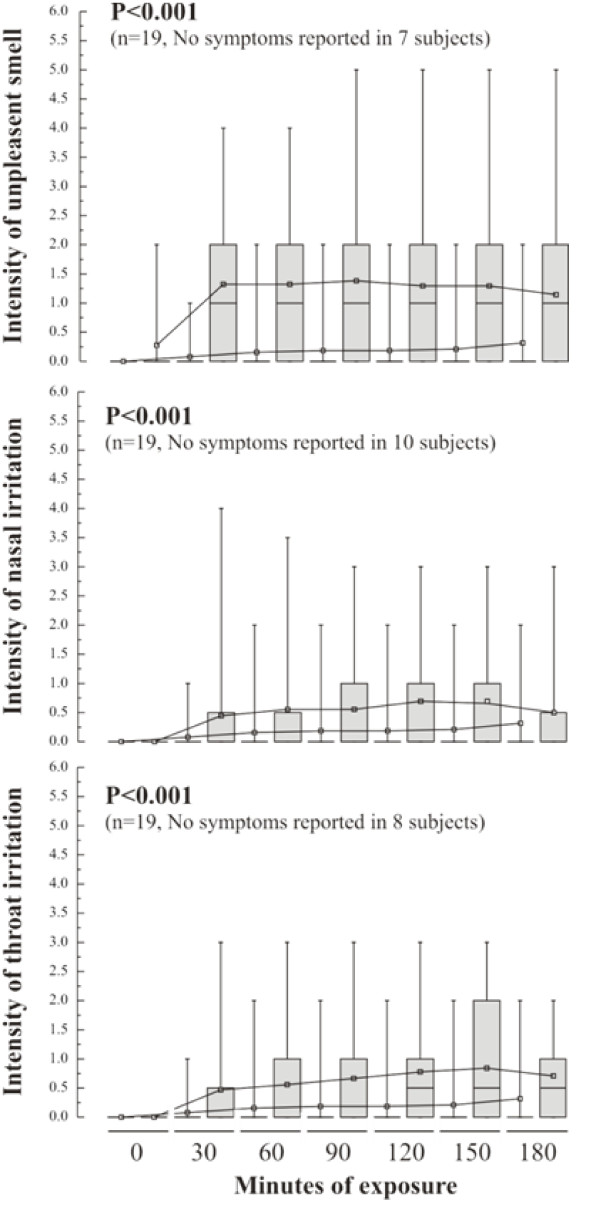
**Intensity of recorded symptoms**. The intensity of the prominent symptoms (unpleasant smell, throat and nasal irritation) observed in subjects exposed to wood smoke (gray box plots) and filtered air. Intensity scores are presented as median values (central line) with the inter-quartile range (box) and 95^th ^percentiles (whiskers). The number of subject exhibiting symptoms is recorded for each of the symptoms, together with the level of significance determined using the Kruskal-Wallis test.

No significant changes were observed in lung function, i.e. vital capacity (VC), forced vital capacity (FVC) and forced expiratory capacity in one second (FEV_1_) or exhaled NO (FE_NO_) at any of the time points examined after the wood smoke challenge (data not shown). Despite this, subjects reported mild but progressive irritation in the nose and throat during the wood smoke exposures (p < 0.001), peaking between 1.5 and 2.5 hours into the exposure, figure 1. Subjects also reported an awareness of an unpleasant smell during the wood smoke exposures (p < 0.001), figure 1. It was notable that a subset of the subjects (7-10 of the 19) exposed to wood smoke reported no symptoms.

There were also no significant changes determined in the differential cell counts in bronchial wash (BW) and bronchoalveolar lavage (BAL) fluid samples obtained 24 hours post air and wood smoke exposures, table 1. The concentration of MPO and MMP-9 were also unaltered in BAL and BW following the wood smoke challenge, table 1.

**Table 1:** Cell counts and inflammatory marker concentrations in BW and BAL fluids

	Bronchial Wash		Bonchoalveolar Lavage	
	
	Post-Air	Post-WS	Post-Air	Post-WS
**Total cells**	7.8 (6.3-11.5)	7.6 (5.7-10.2)	13.3 (8.3-13.7)	11.2 (8.5-14.3)
**Macrophages**	5.65 (3.73-8.53)	5.43 (3.55-7.66)	9.86 (7.28-11.84)	9.07 (6.66-12.87)
**Neutrophils**	1.73 (0.93-3.09)	1.71 (0.97-2.30)	0.25 (0.11-0.54)	0.22 (0.09-0.41)
**Lymphocytes**	0.40 (0.21-0.68)	0.57 (0.21-0.73)	1.29 (0.83-2.08)	1.19 (0.76-1.52)
**Eosinophils**	0.00 (0.00-0.03)	0.01 (0.00-0.03)	0.01 (0.00-0.06)	0.01 (0.00-0.04)
**Mast cells**	1.00 (0.00-6.50)	2.00 (0.00-7.00)	2.00 (0.00-4.00)	2.00 (0.00-5.00)
**MPO ng/ml**	8.62 (5.41-18.00)	6.12 (4.31-10.34)	0.59 (0.32-1.39)	0.53 (0.40-0.92)
**MMP9 ng/ml**	5.99 (3.33-16.11)	4.41 (3.10-6.77)	0.30 (0.24-0.91)	0.27 (0.18-0.48)

Cell counts and inflammatory marker concentrations in BW and BAL fluids obtained from subjects exposed to wood smoke (mean 224 μg/m^3 ^PM_2.5 _for 3 hours) or filtered air with no significant difference in the responses detected. Cell counts are given as cells × 10^4^/ml. All data is expressed as medians with interquartile ranges.

**Figure 2:**
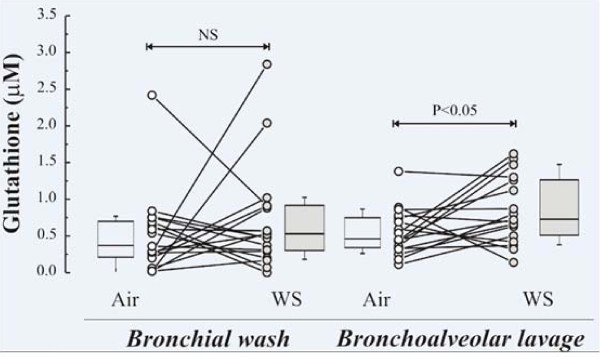
**BAL and BW concentrations of glutathione (GSH)**. Glutathione (GSH) concentrations in BW and BAL fluid samples obtained from subjects 24 hours after exposure to wood smoke (mean 224 μg/m^3 ^PM_2.5_) or filtered air for 3 hours. Individual responses, as well as group medians, with inter-quartile ranges and 95-percentage confidence intervals are illustrated. Comparisons of the concentrations after air versus after wood smoke were performed using the Wilcoxon's Signed Rank test. P values are illustrated where these were significant. Non-significant responses (NS) are also shown.

Of the low molecular weight antioxidants examined in BW and BAL (ascorbate, urate and glutathione) only BAL GSH was found to be significantly increased following wood smoke exposure (p = 0.025), table 2, though a considerable inter-individual variation in response was observed, figure 2. The wood smoke induced increase in lower airway respiratory tract fluid GSH occurred in the absence of increased GSSG concentrations suggesting the absence of oxidative stress at this late time point post-exposure. No wood smoke-induced alteration in low molecular weight antioxidant concentrations was observed in the endobronchial mucosal biopsies, table 3. Neither was there any evidence of an induction of HO-1 protein or increased GST activity post wood smoke, table 3.

**Table 2:** Antioxidant and protein measurements in BW and BAL

	Bronchial Wash		Bronchoalveolar Lavage	
	
	Post-Air	Post-WS	Post-Air	Post-WS
**Total glutathione **(μM)	0.76 (0.35-1.00)	0.94 (0.94-1.14)	**0.73 **(0.38-0.99)	0.87 * (0.64-1.45)
**Glutathione **(μM)	0.36 (0.25-0.75)	0.48 (0.31-0.91)	**0.54 **(0.32-0.74)	0.72 * (0.46-1.19)
**Glutathione disulphide **(μM)	0.11 (0.05-0.30)	0.20 (0.11-0.26)	0.07 (0.00-0.14)	0.04 (0.00-0.13)
**% GSSG**	23.0 (8.8-33.3)	16.9 (9.6-29.9)	7.78 (0.17-22.6)	3.58 (0.00-18.6)
**Total vitamin C **(μM)	0.36 (0.32-0.45)	0.42 (0.32-0.45)	0.50 (0.42-0.56)	0.53 (0.41-0.63)
**Ascorbic acid **(μM)	0.23 (0.21-0.31)	0.24 (0.21-0.32)	0.28 (0.24-0.39)	0.33 (0.13-0.45)
**Dehydroascorbate **(μM)	0.09 (0.05-0.13)	0.12 (0.08-0.15)	0.23 (0.15-0.30)	0.18 (0.15-0.31)
**% oxidised vitamin C**	29.9 (26.6-31.9)	29.2 (24.7-36.7)	33.1 (25.8-46.8)	30.3 (22.3-38.5)
**Urate **(μM)	0.90 (0.80-1.10)	0.86 (0.81-1.01)	0.79 (0.75-1.06)	0.80 (0.73-0.99)
**Total protein **(mg/ml)	0.17 (0.13-0.19)	0.14 (0.13-0.18)	0.14 (0.11-0.16)	0.14 (0.11-0.15)
**Albumin **(μg/ml)	17.0 (14.0-26.0)	18.0 (9.80-35.0)	30.0 (25.0-46.0)	33.0 (24.0-45.0)

Antioxidant and protein measurements in BW and BAL obtained from subjects exposed to wood smoke (mean 224 μg/m^3 ^PM_2.5 _for 3 hours) and filtered air. Data are given as medians with inter-quartile range.

* Significant difference between exposures, p < 0.05

**Table 3:** Endobronchial biopsy concentrations of antioxidants, antioxidant enzyme and protein

	Air	Wood smoke
**Reduced GSH **(μM/mg protein)	25.9 (19.3 - 29.3)	22.8 (20.2 - 27.5)
**% GSSG**	7.4 (4.5-9.9)	9.0 (6.6-15.8)
**Ascorbate **(μM/mg protein)	8.07 (6.35 - 10.1)	8.02 (6.67 - 10.2)
**% DHA**	23.1 (17.5-32.9)	23.0 (15.4-33.0)
**Urate **(μM/mg protein)	1.59 (1.09 - 2.24)	1.80 (0.11 - 2.85)
**HO-1 **(ng/mg protein)	7.32 (4.48 - 9.95)	7.52 (5.34 - 9.89)
**Total Protein **(mg/ml)	0.12 (0.09 - 0.16)	0.12 (0.10 - 0.14)
**Glutathione S-transferase **(U/mg protein)	0.55 (0.39 - 0.82)	0.59 (0.37 - 0.84)

Antioxidant and protein concentrations as well as glutathione S-transferase activity in endobronchial biopsies obtained from subjects exposed to wood smoke (mean 224 μg/m^3 ^PM_2.5 _for 3 hours) and filtered air. No significant differences between air and wood smoke were detected. Data are given as medians with inter-quartile ranges.

Correlation analyses were carried out between post-air GSH concentrations and wood smoke-induced GSH responses.

A significant negative correlation was observed between the baseline GSH concentration and the GSH response to wood smoke, which was stronger in the bronchial wash (ρ = -0.839, p < 0.001) than in the BAL samples (ρ = -0.601, p = 0.011). A similar pattern was observed in biopsy tissues, despite the absence of a significant GSH tissue response: ρ = −0.797, p < 0.001. These data suggest that the greatest induction and mobilisation of GSH to the air-lung interface occurred in those subjects with low baseline GSH concentrations.

## Discussion

The present study investigated the impact of a relatively high dose of wood smoke-derived particulate matter (224 μg/m^3 ^PM_2.5 _for 3 hours) on airway function, inflammation and the endogenous antioxidant defences at the air-lung interface. Exposure to wood smoke resulted in significantly increased levels of reduced glutathione in bronchoalveolar lavage fluid from healthy subjects sampled 24 hours after exposures, together with increase in mucosal symptoms. There were no changes in BAL cell numbers, mediator concentrations, lung function or exhaled NO.

The current findings contrast markedly with the pattern of response observed in subjects exposed to diesel exhaust PM, at a lower total dose (PM_10 _300 μg/m^3 ^for 1 hour or 100 μg/m^3 ^for 2 hours). Proximal airway neutrophilia, lymphocytosis and mastocytosis was clearly present 6-18 hour post exposure, associated with an up-regulation of redox sensitive signalling and EGFR pathways in the bronchial mucosa together with increased GSH concentrations in BAL fluid [25-29,39-41]. The series of diesel exhaust studies in healthy subjects, and individuals with respiratory and cardiovascular disease, clearly support the notion of diesel exhaust to cause wide spread inflammatory events and adverse cardiovascular consequences [42-44].

The results of this study indicate that wood smoke, at least from the presently investigated exposure situation, is relatively non-toxic, as no significant inflammatory responses or consumption of antioxidants at the airway surface were demonstrated.

Wood smoke and other biomass source-derived PM samples can have some general compositional similarities with diesel exhaust-derived particulates, but in several aspects the physico-chemical properties differ. Diesel exhaust particles mainly consist of two particle types: soot agglomerates coated with condensed matter (e.g. hydrocarbons and metallic ash) and volatile nuclei mode particles with a high fraction of organics [45]. The knowledge of the properties of wood smoke particles is less advanced but the particles appear more heterogeneous, as highlighted when comparing different combustion systems and combustion phases [3]. Wood smoke typically contains a substantially higher fraction of water soluble alkali salts and the organic fraction is markedly different, containing higher oxygen content (e.g. monosacharides and methoxyphenols). The trace element composition is also different with wood smoke typically being enriched in Zn. Finally, the properties of the soot core (e.g. primary particle diameter, number of primary particles and soot structure) may also be different. Therefore, it can be expected that magnitude and perhaps also the mechanisms of particle induced toxicity may be markedly different for wood smoke compared to diesel exhaust. Based on the presently investigated biomass and combustion characteristics, wood smoke appears to be less toxic to the airway than diesel exhaust challenge, as reflected by a series of preceding investigations. This conclusion should however be tempered by the knowledge that the organic and elemental composition of PM from biomass combustion varies significantly depending on combustion conditions and fuel type [1,3]. We therefore acknowledge the possibility that other combustion situations potentially could yield particles with higher toxicity.

The only previous wood smoke exposures in healthy subjects employed a batch fired wood log stove at a broadly equivalent dose to the present study [22,23]. In many aspects the conditions employed in the two studies were similar (summarised in table 4) though the earlier exposure campaign reported considerably higher number concentration of ultra fine particles which may have played a role for partly differing outcomes between the studies.

**Table 4:** Comparison of the exposure conditions between the present study and previous studies by Barregård and Sällsten

Parameter	Unit	Present study	**Preceding wood smoke exposures **[22-24]
Temperature in chamber	°C	21-24	23
Relative humidity in chamber	%	20-50	35
Exchange rate of air in chamber	times/hour	~10	~2
NO_x _(approximately 90% as NO)	ppm	0.2-0.4	0.6 and 0.7
CO	ppm	5-15	13 and 9.1
1.3-Butadiene	μg/m^3^	11.0 ± 2.8	~6 and ~4
Benzene	μg/m^3^	81.8 ± 14.3	~30 and ~20
PM_2.5 _mass concentration (filter)	μg/m^3^		242 ± 8.2 and 272 ± 2
PM_2.5 _mass concentration (TEOM)	μg/m^3^	224 ± 22	~130-230
Mass median diameter (MMD_a_)	μm	0.218 and 228*	
Particle number concentrations	#/cm^3^	6.7 ± 0.9×10^4^	18.0×10^4 ^and 9.5×10^4^
Geometric mean diameter (GMD)	μm	0.120 ± 0.018**	0.042 and 0.112***
Geometric standard deviation (σ_g_)		1.81 ± 0.06	1.7 and 1.4
Organic carbon (OC × 1.4) fraction	% (of PM_tot_)	~60	
Elemental carbon (EC) fraction	% (of PM_tot_)	~25	
Black smoke (BS) fraction	% (of PM_tot_)		~30 and ~40
Total PAH concentration	μg/m^3^	0.76 ± 0.17****	
		0.68 ± 0.15*****	0.44 and 0.47*****
Semi-volatile PAH fraction	%	74-88	
PM-associated PAH fraction	%	12-26	
Inorganic major ions (alkali salts)	% (of PM_tot_)	~13	~4-5
Trace metals	% (of PM_tot_)	~2	~2

* 2 of 13 exposure occasions

** measured with SMPS (i.e. given as equivalent mobility diameter)

*** measured with ELPI (i.e. given as aerodynamic particle diameter)

**** includes 45 specific PAH compounds (≥ 3 aromatic rings)

***** includes 13 specific (same) PAH compounds (≥ 3 aromatic rings)

In contrast to previous animal and cell culture studies employing wood smoke exposures in which oxidative stress has observed [16,46,47], the present study failed to demonstrate any evidence of decreased antioxidant concentrations in the respiratory tract lining fluid or increased concentrations of GSSG. In addition we observed no evidence of an up-regulation of HO-1 or GST in the bronchial tissues of subjects post wood smoke, which has previously been observed in cell models challenged with PM reflecting protective adaptation to the induction of oxidative stress [48]. The only wood smoke-induced response observed was an increase in BAL GSH, and we acknowledge the possibility of a chance finding due to multiple analyses. We do, however, interpret this as a mobilisation of this antioxidant to the distal lung respiratory tract lining fluids. Increases in BAL GSH have been observed in response to other oxidative challenges [49-51], however the source of the additional GSH has never been identified. Transcriptional regulation of the rate-limiting enzymes involved in GSH synthesis are known to be redox-sensitive [52,53]. Therefore, this response may reflect a protective increase in de novo GSH synthesis and regulated transport out of the cell. However, GSH efflux from cells has also been demonstrated to occur in apoptotic cells before any plasma membrane leakage begins [54]. It is therefore possible, that the observed GSH increase may be an early marker of apoptosis, although this remains to be confirmed.

An additional aspect to consider is our recent findings of differences in deposition in the respiratory tract of wood smoke and motor engine particles in traffic [55,56]. Deposition in the respiratory tract is primarily dependent upon particle size, composition, hygroscopic properties and individual parameters such as breathing pattern and airway geometry. Wood smoke particles generated under the same conditions as in this study had, because of their size distribution and hygroscopicity, a low probability of depositing in the respiratory tract. At similar inhaled mass concentration the deposition of motor exhaust particles in a traffic environment was shown to be 16 times higher by particle number and 3 times higher by surface area. It has been demonstrated that there is a correlation between the toxicity of insoluble submicrometer particles and their total deposited surface area [57]. Thus, it is probable that some of the differences in health response may be explained by variations in respiratory tract deposition.

Taken together, the present findings indicate a potential mobilisation of glutathione to the air-lung interface in the absence of inflammatory cell recruitment into the airways, oxidative stress or induction of mucosal xenobiotic enzymes (HO-1 and GST). This is interpreted as an early response to PM, inferring a refinement to the hierarchical response model which has been employed to characterise the responses of cells to PM challenge and oxidative stress [48,58]. We propose an initial airway tolerance/protection against oxidative stress related to endogenous antioxidant defences in the RTLFs, and speculate on an early antioxidant mobilisation to the air way surface to prevent damage to the underlying epithelium. The response observed in the present study may therefore be considered as adaptive occurring before the triggering of protective antioxidant/xenobiotic enzyme expression under the regulation of Nrf2 [47,57]. It is possible that other wood combustion situations that generate different emission characteristics would yield more pronounced effects, consistent with the responses observed in *in-vitro *models [3], but this remains to be tested under human challenge conditions.

## Conclusions

The current study demonstrated that the healthy airway is able to tolerate a relatively high dose exposure to wood smoke PM derived from the investigated combustion situation. The only significant biological response observed was an increase in GSH in the respiratory tract lining fluids of the distal lung. As this was observed, in the absence of inflammation or the induction of xenobiotic or antioxidant enzymes, we suggest that this represents an early adaptive response, mobilising GSH to the air lung interface. The impact of other wood smoke and biomass combustion situations as well as responses in individuals with pre-existing respiratory and cardiovascular conditions remain to be determined in order to widen the perspectives of biomass combustion and health.

## Abbreviations

PM_2.5_: particulate matter with an aerodynamic diameter of < 2.5 μm; PM_10_: particulate matter with an aerodynamic diameter of < 10 μm; NO: nitric oxide; FE_NO_: fraction of exhaled nitric oxide; EBC: exhaled breath condensate; BW: bronchial wash; BAL: bronchoalveolar lavage; PAH: polycyclic aromatic hydrocarbons; TEOM: tapered element oscillating microbalance; VC: vital capacity; FVC: forced vital capacity; FEV_1_: forced expiratory capacity in one second; MPO: myeloperoxidase; MMP-9: matrix metalloproteinase 9; GSH: glutathione; GSSG: glutathione disulphide; DTNB: 5,5'-dithio-bis(2-nitrobenzoic acid), Ellman's reagent; HO-1: heme oxygenase 1; GST: glutathione S transferase

## Competing interests

The authors declare that they have no competing interests.

## Authors' contributions

**MS**: study design, statistical analysis, evaluation of data, manuscript preparation. **RD**: performed the antioxidant measurements, data analysis, manuscript writing. **CB**: study design, responsible for exposure and combustion set-ups, manuscript preparation, PM measurements and characterisation, data evaluation, scientific contribution. **JPa**: selection and adaption of PM methods, performed PM measurements and characterisation, aerosol selection and generation, exposure chamber evaluation, manuscript contribution. **ES**: study design, PM measurements and characterisation, scientific contribution. **JL**: PM measurements and characterisation, exposure chamber evaluation, manuscript contribution. **RW**: chemical characterisations, manuscript contribution. **JB**: study planning, subject inclusions and medical responsibility during exposures, manuscript contribution. **SB**: study planning, subject inclusions, bronchoscopy and manuscript contribution. **AFB**: study planning, subject inclusions, bronchoscopy, manuscript contribution. **JPo**: study design, biomedical analyses, scientific and manuscript contributions **TS**: study design, scientific and manuscript contributions. **ISM**: study design, responsible for the antioxidant measurements, data analysis, and manuscript preparation. **AB**: study design, subject inclusions, bronchoscopies, data analyses, scientific and manuscript contribution. All authors have read and approved the final manuscript.
